# IGF2BP3 facilitates cell proliferation and tumorigenesis via modulation of JAK/STAT signalling pathway in human bladder cancer

**DOI:** 10.1111/jcmm.16003

**Published:** 2020-10-22

**Authors:** Wei Huang, Yuanyuan Li, Cheng Zhang, Huihai Zha, Xiaocheng Zhou, Bin Fu, Ju Guo, Gongxian Wang

**Affiliations:** ^1^ Department of Urology The First Affiliated Hospital of Nanchang University Nanchang China; ^2^ Department of Gastroenterology The First Affiliated Hospital of Nanchang University Nanchang China

**Keywords:** bladder cancer, IGF2BP3, JAK/STAT, prognosis, proliferation

## Abstract

Insulin‐like growth factor‐2 messenger RNA‐binding protein 3 (IGF2BP3) has been reported to contribute to tumorigenesis in several human cancers. However, the biological functions of IGF2BP3 in bladder cancer are poorly understood. We investigated the relation between IGF2BP3 expression and prognosis of bladder cancer patients. Cell proliferation, cell cycle and cell apoptosis assays were performed to assess IGF2BP3 functions. The results showed that IGF2BP3 was overexpressed in bladder cancer tissues compared with that in normal bladder tissues, and its higher expression was closely correlated with poor prognosis in bladder cancer patients. Overexpression of IGF2BP3 markedly promoted cell proliferation and cell cycle progression and inhibited cell apoptosis, while knockdown of IGF2BP3 notably suppressed the proliferation, promoted cell apoptosis and induced cell cycle arrest at the G0/G1 phase. Mechanistically, we revealed that IGF2BP3 promotes the activation of the JAK/STAT pathway in bladder cancer cells. Moreover, the JAK/STAT inhibitor dramatically blocked the tumour‐promoting activity of IGF2BP3. Tumour growth in vivo was also suppressed by knocking down of IGF2BP3. Hence, IGF2BP3 facilitated bladder cancer cell proliferation by activating the JAK/STAT signalling pathway. These findings suggest that IGF2BP3 exhibits an oncogenic effect in human bladder cancer progression.

## 1 INTRODUCTION

As one of the most common malignancies of the urinary tract, bladder cancer is the 11th most frequently diagnosed cancer worldwide.^1^ Currently, there are approximately 81 190 new cases per year in the United States and 17 240 people die from the disease annually.^2^ Although there have been significant advancements in the development of surgical techniques and adjunct treatment, the prognosis of bladder cancer remains poor. Therefore, it is essential to understand the molecular mechanism in order to explore effective diagnostic and prognostic markers in bladder cancer.

As a messenger RNA (mRNA) binding protein, the insulin‐like growth factor‐2 messenger RNA‐binding protein 3 (IGF2BP3) is a member of the IGF2 mRNA‐binding protein family and plays significant roles involved regulating the translation of insulin‐like growth factor‐2.^3^, ^4^, ^5^ Previous research reported the oncofoetal protein IGF2BP3 participates in RNA trafficking and stabilization and cell proliferation and cell migration in the early processes of embryogenesis.^6^, ^7^ Many studies indicate that IGF2BP3 is highly expressed in a variety of tumour tissues compared to adjacent normal tissues, including lung cancer,^8^ gastric cancer,^9^ pancreatic cancer,^10^ kidney cancer,^11^ suggesting a tumour‐promoting role of IGF2BP3.^12^ High IGF2BP3 expression predicts metastasis formation and poor survival in renal cell carcinoma.^13^ Moreover, increased IGF2BP3 expression facilitates the aggression of colorectal cancer cells via regulating epithelial‐mesenchymal transition.^14^ Recently, a study reported that IGF2BP3 was directly associated with a deubiquitinase named Ubiquitin‐specific peptidase 10 and attenuated its function in stabilizing p53 protein in lung cancer.^15^ However, little is known about the function of IGF2BP3 in bladder cancer.

In this study, we explored the expression of IGF2BP3 in bladder cancer and subsequently investigated the prognostic and molecular function of IGF2BP3. Our study showed that IGF2BP3 could be a prognostic factor in bladder cancer and serve as a potential target for new therapeutic strategies.

## 2 MATERIALS AND METHODS

### 2.1 Data mining

The data for mRNA expressions (mRNA SeqV2) and follow‐up data of human bladder cancer were obtained from the Cancer Genome Atlas database (TCGA, https://tcga‐data.nci.nih.gov/tcga). The gene expression profile was extracted from TCGA RNA‐seq data, which contained 267 primary bladder cancer tissues and 19 surrounding non‐cancer tissues. All profile data were analysed using R statistical environment and further calculated. Kaplan‐Meier survival analysis was performed to validate the prognostic value of IGF2BP3 in bladder cancer. To gain further insight into the biological processes/signalling pathway and phenotypes of IGF2BP3 involved in bladder cancer pathogenesis, a gene set enrichment analysis (GSEA, version 2.2.3) was performed. IGF2BP3 expression is first dichotomized as low and high categories to annotate phenotypes. GSEA of genes' correlations with the phenotypes is further tested by using C2 set from MSigDB (http://software.broadinstitute.org/gsea/msigdb). The gene sets that are significantly enriched by the genes associated with high expression of IGF2BP3 [false discovery rate (FDR) <0.25] were selected as enriched gene sets.

### 2.2 Patients and immunohistochemistry in bladder specimens

Two 50‐spot, paraffin‐embedded, human bladder cancer tissue microarrays (HBlaU050CS01) containing 40 bladder tumour and 10 adjacent bladder non‐tumour tissues were purchased (Outdo Biotech, Shanghai, China). A total of 100 primary bladder cancer samples from 2013 to 2018 were enrolled in this study from the Department of Urology, The First Affiliated Hospital of Nanchang University. The clinicopathological information of these patients is summarized in Table 1. The study was approved by the Ethics Committee of The First Affiliated Hospital of Nanchang University and informed consent was obtained from all patients. All resected bladder tissues were fixed in 10% formaldehyde for 48 hours and embedded in paraffin. Then, paraffin‐embedded tissues were cut into 5 µm thick sections. Slides were deparaffinized with xylene and rehydrated with graded alcohol for pre‐treatment. Antigen retrieval was performed by microwave heating with citric acid buffer. To block endogenous peroxide, slides were incubated with 3% H_2_O_2_ for 10 minutes in a wet box. Then, slides were incubated with primary antibodies anti‐IGF2BP3 (Abcam, Cambridge, MA) at a dilution of 1:100 overnight at 4°C in a humidified box. After washing with phosphate‐buffered saline, slides were incubated with a secondary HRP‐labelled anti‐rabbit antibody (1:50; Beyotime, Shanghai, China) for 30 minutes at room temperature. Tissue sections were stained with 3,3′‐diaminobenzidine (DAB) and counterstained with haematoxylin. Images were acquired with a Nikon microscope camera (Nikon Americas Inc, NY).

**TABLE 1:** Clinicopathological characteristics of patients with bladder cancer

Characteristics	Number of cases
Age, years	
≥60	72
<60	28
Gender	
Male	64
Female	36
T classification	
Ta	14
T1	12
T2	40
T3	25
T4	9
N classification	
N0	87
N1	7
N2	5
N3	1
Metastasis	
No	99
Yes	1
Tumour grade	
PUNLMP	1
Low grade	30
High grade	69
Expression of IGF2BP3	
Low expression	35
High expression	65

Abbreviation: PUNLMP, papillary urothelial neoplasm of low malignant potential

### 2.3 Cell lines and culture

A normal human bladder uroepithelium cell line (SV‐HUC‐1) and four bladder cancer cell lines (5637, J82, T24 and UMUC3) were obtained from The Cell Bank of Chinese Academy of Sciences (Shanghai, China). SV‐HUC‐1, T24 and UMUC3 were cultured in DMEM (Gibco, Carlsbad, CA). 5637 and J82 were cultured in RPMI‐1640 medium (Gibco, Carlsbad, CA). All culture media contained 1% penicillin/streptomycin and 10% foetal bovine serum (FBS, Gibco). The cells were incubated in humidified air at 37°C with 5% CO_2_.

### 2.4 Lentivirus construction and infection

Three human siRNA sequences (siIGF2BP3‐1, 5’‐ GCAAAGGATTCGGAAACTT‐3’, siIGF2BP3‐2, 5’‐ GGTGAAACTTGAAGCTCAT −3’, siIGF2BP3‐3, 5’‐ CCAGACACCTGATGAGAAT −3’) and negative control siRNA (siNC) were cloned in pLKO.1‐puro vector to endogenously down‐regulate IGF2BP3. The coding sequences (CDS) region of human IGF2BP3 was synthesized and cloned into pLVX‐puro vector to overexpress IGF2BP3. The synthesized core plasmid was confirmed by DNA sequencing (Majorbio, Shanghai, China). For lentiviral production, 293T cells were transfected with the lentiviral vector along with packaging plasmids using Lipofectamine 2000 (Invitrogen, Shanghai, China). Culture media was collected, pooled and filtered at 48 hours and 72 hours after transfection. Then, the indicated lentivirus was used to infect the bladder cancer cell lines, and the expression of IGF2BP3 was evaluated by real‐time quantitative PCR and Western blotting.

### 2.5 Quantitative real‐time polymerase chain reaction (QRT‐PCR)

Total RNA was extracted following the manufacturer's protocol with TRIzol reagent (Invitrogen, Carlsbad, CA), dissolved in RNA‐free H_2_O and stored at −80°C. cDNA synthesis was performed from each 1 μg RNA sample using the Reverse Transcriptase Kit (Thermo, USA). Then, qPCR was performed on a real‐time detector (ABI, USA) using a SYBR Green PCR kit (Thermo, USA). Primer sequences of IGF2BP3 were as follows: primer F, 5'‐ GCACTTCCCTTTGTTGTAGTC −3', primer R, 5'‐ AGCACTTCCCTTAGGTTACTC −3'. Expression data were calculated using the 2^‐ΔΔCt^ method and normalized by taking GAPDH as an internal reference to control the relative expression levels.

### 2.6 Western blotting

For Western blotting, cells were lysed with RIPA lysis buffer kit (Jrdun Biotechnology, CA), supernatants were collected after spin and total proteins were measured using the BCA protein quantification kit (Thermo Scientific, USA). Total protein samples were separated by 10% or 15% sodium dodecyl sulphate polyacrylamide gel electrophoresis (SDS‐PAGE). Then, the samples were transferred onto nitrocellulose membranes. After blocking with 5% fat‐free milk for 1 hour at room temperature, the membranes were incubated with primary antibodies against IGF2BP3 (1:1000; Abcam, USA), CyclinD1 (1:1000; Cell Signaling, Germany), p‐STAT3 (1:5000, Abcam, USA), STAT3 (1:2000, Abcam, USA), BAX (1:1000; Abcam, USA), Bcl2 (1:500, Abcam, USA) and GAPDH (1:2000; Cell Signaling, Germany) overnight at 4°C. Following three washes with TBST buffer, the membranes were incubated with secondary goat anti‐rabbit antibodies conjugated with HRP for 1 hour at room temperature. Signals were visualized using the enhanced chemiluminescence kit (Millipore, USA) and detected by the Tanon‐5200 Imaging system. Integrated relative densities of individual bands were quantified using Image J (National Institutes of Health, Bethesda, MD).

### 2.7 Cell proliferation

Cell proliferation was tested using the Cell Counting Kit‐8 (CCK‐8, Beyotime, Shanghai, China). Each well of a 96‐well culture plate was seeded with approximately 3 × 10^3^ target cells and maintained at 37℃ overnight. At 0, 12, 24, 48 and 72 hours after transfection, cells were treated with CCK‐8 (10 µl/well) and incubated for 1 hour. Subsequently, the optical density of each well was read at 450 nm. Every sample was assayed three times.

### 2.8 Cell cycle assay

Cells were collected, washed and fixed in 70% ethanol. The fixed cells were subsequently washed in PBS, incubated with RNAase and stained with 50 μg/ml propidium iodide at 37°C for 30 min. Flow cytometric analysis was performed using a FACSCalibur instrument (Becton‐Dickinson, San Jose, CA), and the results were analysed using FlowJo7.6 software.

### 2.9 Cell apoptosis assay

Apoptotic cells were detected using Annexin V‐FITC/PI apoptosis detection kit (Beyotime, Shanghai, China). Briefly, cells were collected with trypsin/EDTA and washed by ice‐cold PBS and then resuspended in binding buffer. Next, cells were incubated with 5 µl Annexin V‐ FITC at 4°C for 15 minutes and 5 µl PI at 4°C for 5 minutes in a dark place. After incubation, the apoptotic cells were quantified by flow cytometry (BD Biosciences), and data were analysed with BD AccuriTM C6 software.

### 2.10 Transwell migration and invasion assay

Transwell migration and invasion assays were performed using Transwell chambers (Costar Corporation, MA, USA) without or with Matrigel (Corning, Kennebunk, USA). 0.7 ml of 10% FBS‐containing medium was placed to the lower chamber, in which 3 × 10^4^ cells suspended in 300 µl of serum‐free medium were seeded in the upper chamber for 24 hours. Cells were fixed with 4% formaldehyde for 10 minutes, and then, we removed the adhering cells, followed by washing once with PBS. Subsequently, 1 ml/well 0.5% crystal violet was used to stain the cells for 30 minutes, followed by washing with PBS three times. The migrating or invading cell numbers were counted by a 200 × microscope.

### 2.11 Xenograft tumour model

Male BALB/c nude mice (four to six weeks old), weighting 18‐20 g, were purchased from Shanghai Experimental Animal Center (Shanghai, China). All mice were kept in a strict pathogen‐free condition. The Ethics Committee for Animal Experiments of The First Affiliated Hospital of Nanchang University approved the animal experiments. We divided the twelve mice into two groups: a control group (siNC transfected cells) and a siIGF2BP3 group. To establish the xenograft model, a total of 4 × 10^6^ tumour cells were subcutaneously injected into the right flank of the nude mice. Every three days, we measured the tumour length and width with caliper. Tumour volume was calculated using the formula: (length* width^2^)/2. At the end‐point, the mice were euthanized, and tumour tissues were weighted.

### 2.12 Statistical analysis

All data were represented as means ± standard deviations (SD). All differences between two independent groups were analysed using a two‐tailed Students’ *t* test. All differences between multiple groups were analysed using ANOVA tests. Survival data were analysed by univariate and multivariate Cox regression analyses. Survival curves were analysed using the Kaplan‐Meier method and compared using the log‐rank test. SPSS version 16.0 software (IBM Corporation, USA) was used for statistical analysis. A *P*‐value <0.05 was considered statistically significant.

## 3 RESULTS

### 3.1 Up‐regulation of IGF2BP3 in bladder cancer

**FIGURE 1:**
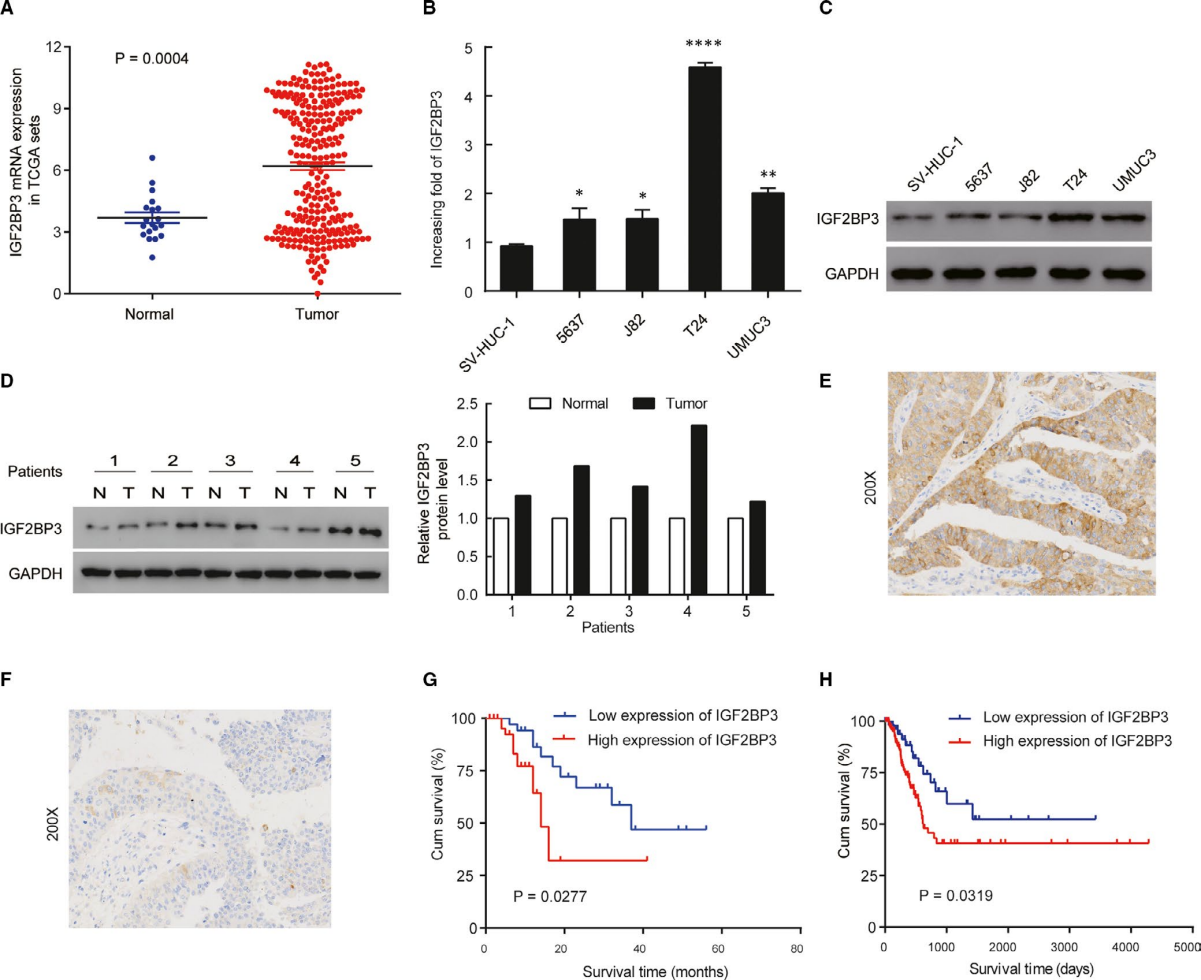
IGF2BP3 is up‐regulated in bladder cancer tissues, and this up‐regulation is associated with poor prognosis. A, Comparison of IGF2BP3 expression of bladder cancer patients in the TCGA profile. B, qRT‐PCR analysis of IGF2BP3 expression in SV‐HUC‐1 and bladder cancer cell lines. C, Western blotting of IGF2BP3 expression in SV‐HUC‐1 and bladder cancer cell lines. D, IGF2BP3 protein expression level in five paired bladder cancer tissues. E, Representative images of IGF2BP3 high expression in bladder cancer. F, Representative images of IGF2BP3 low expression in bladder cancer. G, Patients with high IGF2BP3 expression level had a shorter overall survival compared with that of the low IGF2BP3 expression level group. H, Kaplan‐Meier curves of overall survival of bladder cancer patients in the TCGA profile. *P < .05, **P < .01, ****P < .0001

To explore the tumour‐promoting or tumour‐suppressing effect of IGF2BP3 on bladder cancer, the expression of IGF2BP3 was evaluated in bladder cancer tissues and normal tissues from TCGA datasets. As shown in Figure 1A, the expression of IGF2BP3 in bladder cancer tissues was significantly higher than that in normal tissues (*P* = 0.0004); the means ± SD for IGF2BP3 expression in normal tissues and bladder cancer were 3.687 ± 0.258 and 6.195 ± 0.187, respectively. Real‐time PCR and Western blotting showed higher IGF2BP3 in all four bladder cancer cell lines (5637, J82, T24 and UMUC3) compared to normal human bladder uroepithelium cell line (SV‐HUC‐1) (Figure 1B and C). Furthermore, we assessed the expression of IGF2BP3 with Western blotting in 5 pairs of bladder cancer and matched normal adjacent tissues. Compared with adjacent noncancerous tissues, we found that the protein levels of IGF2BP3 were higher in bladder cancer tissues (Figure 1D). In addition, we also evaluated the expression of IGF2BP3 by immunohistochemical analysis of 100 patients who underwent transurethral resection of the bladder or radical cystectomy. High expression of IGF2BP3 was observed in the tumour cells of 65% of the patients. As shown in Figure 1E and F, IGF2BP3 was expressed at higher levels in advanced stage tumours (T2‐T4) (Figure 1E) and at lower levels in early stage tumours (Ta‐T1) (Figure 1F).

### 3.2 High igf2bp3 expression in bladder cancer correlates with poor survival

The association between IGF2BP3 expression and the clinicopathological features of bladder cancer was shown in Table 2. Overexpression of IGF2BP3 was observed to be significantly associated with T classification (*P* = 0.026) and tumour grade (*P* = 0.001). Yet, the IGF2BP3 level was not associated with gender, age, N classification and metastasis. As shown in Table 3, Univariate Cox regression analysis confirmed that higher IGF2BP3 expression (*P* < .001), T classification (*P* < .001) and tumour grade (*P* = 0.002) were associated with an increased risk of death. Multivariate Cox regression analysis revealed that IGF2BP3 level (HR = 4.583, 95% CI 2.242‐8.662, *P* < .001) and T classification (HR = 1.39, 95% CI 1.051‐1.837, *P* = 0.021) could be independent prognostic factors for overall survival (OS). The OS rate of patients with bladder cancer with higher IGF2BP3 expression in tumours was significantly poorer than that of patients with lower IGF2BP3 expression in tumours (*P* = 0.0277, Figure 1G). That was consistent with the Kaplan‐Meier analysis of TCGA which revealed a negative correlation of IGF2BP3 expression with the OS (*P* = 0.0319, Figure 1H). Hence, these findings suggest that IGF2BP3 is highly expressed in bladder cancer and that IGF2BP3 is a factor for predicting poor survival in patients with bladder cancer.

**TABLE 2:** Relationship between IGF2BP3 expression and clinicopathological features in patients with bladder cancer

Characteristics	IGF2BP3		*P* value
	Low expression, no	High expression, no	
Age, years			
≥60	22	50	.135
<60	13	15	
Gender			
Male	23	41	.793
Female	12	24	
T classification			
Ta	9	5	.026
T1	6	6	
T2	14	26	
T3	4	21	
T4	2	7	
N classification			
N0	31	56	.476
N1	2	5	
N2	1	4	
N3	1	0	
Metastasis			
No	35	64	.461
Yes	0	1	
Tumour grade			
PUNLMP	1	0	.001
Low grade	18	12	
High grade	16	53	

**TABLE 3:** IGF2BP3 regression analysis for predicting cancer specific survival of bladder cancer

	Univariate analysis			Multivariate analysis		
	*P* value	Hazard ratio	95% confidence interval	*P* value	Hazard ratio	95% confidence interval
IGF2BP3	<.001	4.942	2.851‐8.567	<.001	4.583	2.242‐8.662
T classification	<.001	1.599	1.286‐1.987	.021	1.39	1.051‐1.837
Tumour grade	.002	2.358	1.38‐4.028	.981	0.991	0.467‐2.102

### 3.3 IGF2BP3 enhanced proliferation of bladder cancer cells

**FIGURE 2:**
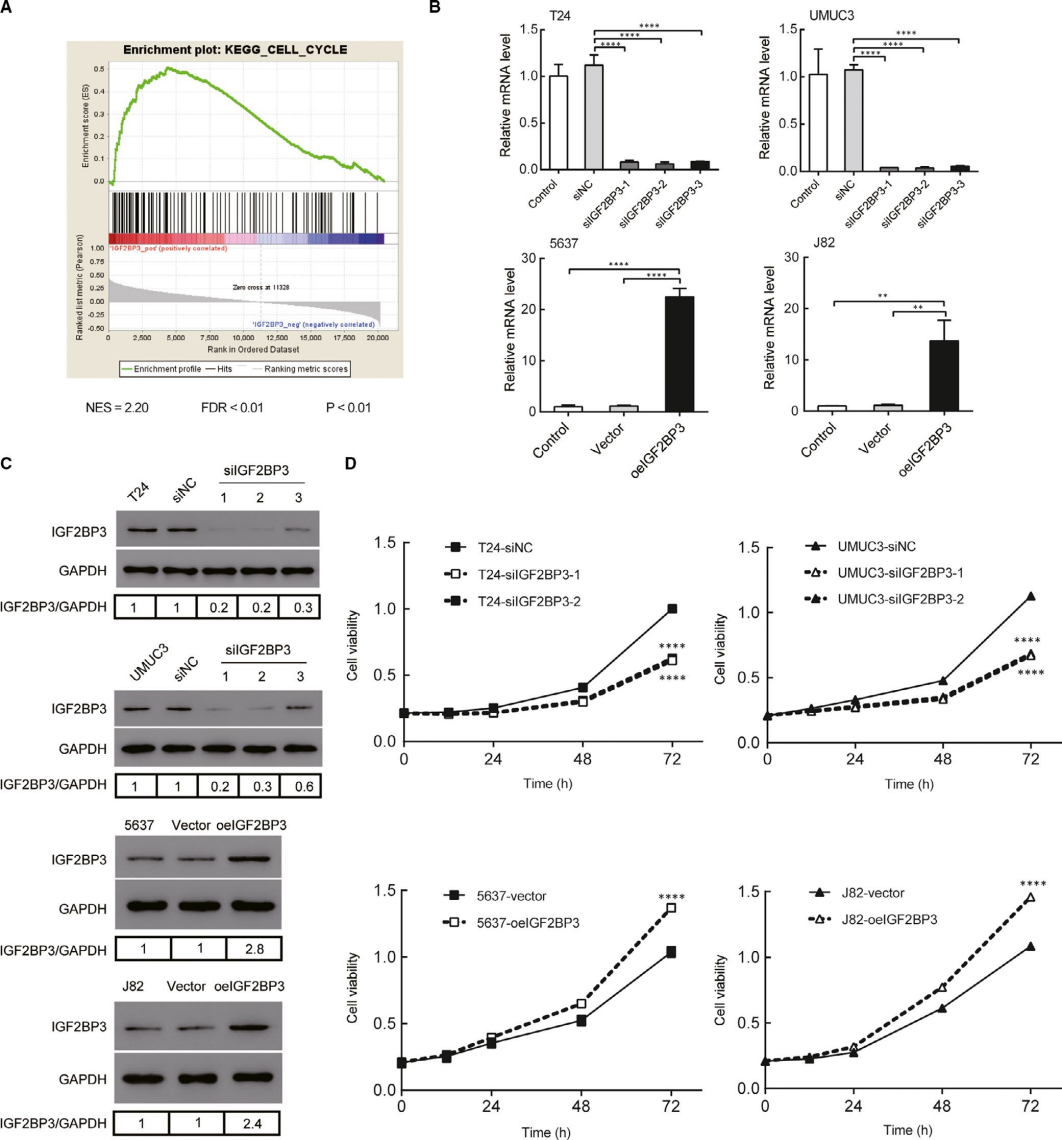
IGF2BP3 enhances proliferation of bladder cancer cells. A, GSEA plot showing that IGF2BP3 expression positively correlated with cell cycle activated gene signatures. B and C, IGF2BP3 was stably overexpressed in 5637 cells and J82 cells, silenced in T24 cells and UMUC3 cells by transfection and selection, respectively. IGF2BP3 expression was confirmed by real‐time PCR and Western blotting analysis. D, CCK‐8 assays revealed that down‐regulation of IGF2BP3 inhibited the growth rate of T24 and UMUC3 cells and overexpression of IGF2BP3 promoted the growth rate of 5637 and J82 cells. ****P < .0001

GSEA analysis was performed in the TCGA database and the results showed that higher levels of IGF2BP3 are positively associated with an enrichment of cell cycle gene signatures (Figure 2A). To further explore the biological function of IGF2BP3 in bladder cancer, three IGF2BP3‐specific shRNAs were used to construct IGF2BP3 knockdown cells in T24 and UMUC3 cell lines, which had relatively higher IGF2BP3 expression, and IGF2BP3 was ectopically overexpressed in 5637 and J82 cell lines, which displayed relatively lower IGF2BP3 expression. The expression of IGF2BP3 was significantly decreased in T24 and UMUC3 cells and overexpressed in 5637 and J82 cells both at mRNA and protein level (Figure 2B and C). CCK‐8 assays showed silencing of IGF2BP3 using siIGF2BP3‐1 and siIGF2BP3‐2 significantly reduced cell viability of T24 and UMUC3 cells (Figure 2D). Furthermore, overexpression of IGF2BP3 significantly promoted cell viability of 5637 and J82 cells, which were approximately 1.0‐fold higher than that of vector control cells at 72 hours after transfection (Figure 2D). Collectively, these results indicate that IGF2BP3 promotes the proliferative ability of bladder cancer cells.

### 3.4 IGF2BP3 is involved in cell cycle G1 to S phase transition in bladder cancer cells

**FIGURE 3:**
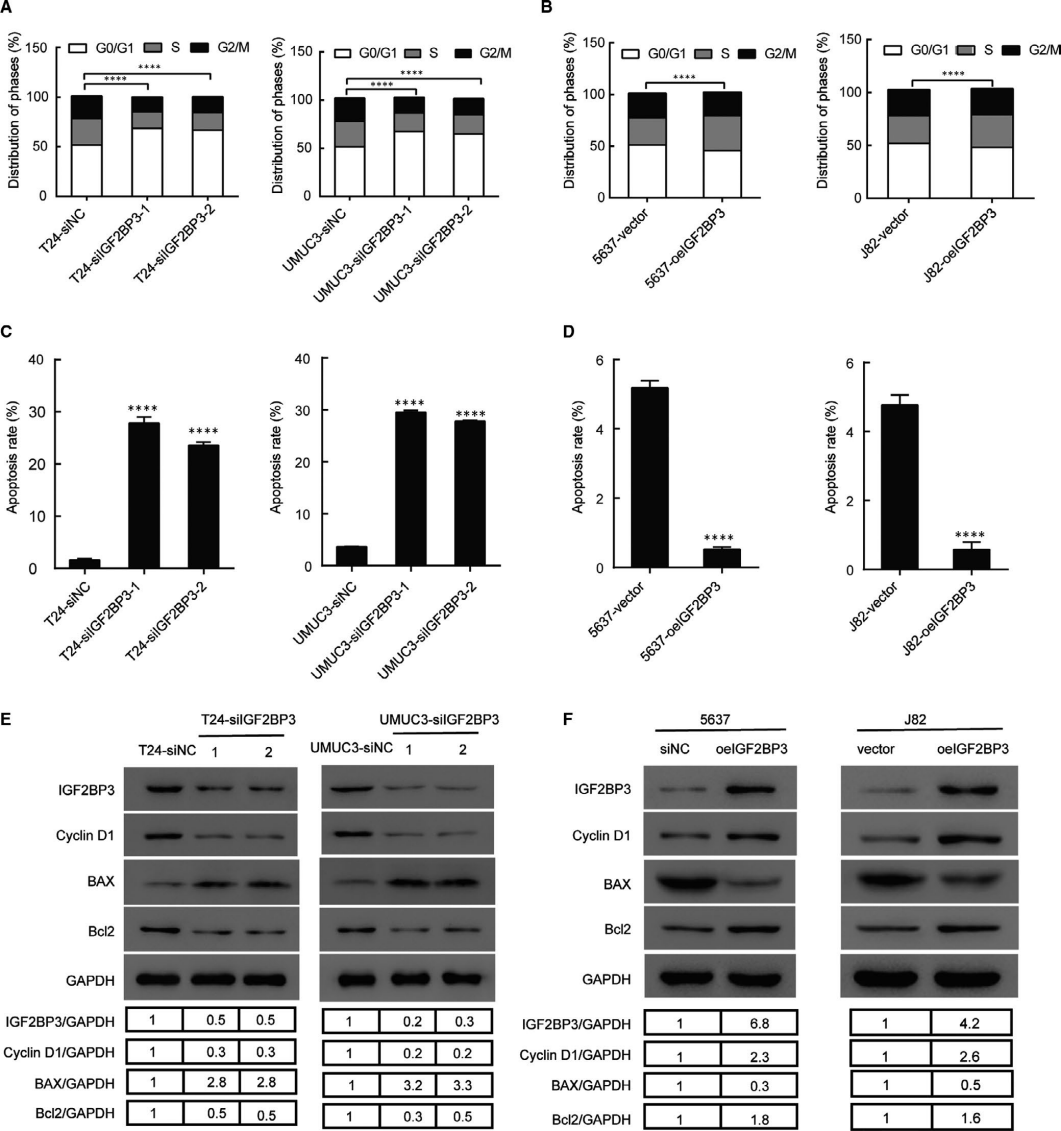
IGF2BP3 is involved in cell cycle progression and interfered with cell apoptosis. A, Down‐regulation of IGF2BP3 induced G0/G1 phase arrest in T24 and UMUC3 cells. B, Up‐regulation of IGF2BP3 promoted cell cycle G1/S phase transition in 5637 and J82 cells. C, Down‐regulation of IGF2BP3 promoted the T24 and UMUC3 cells apoptosis. D, Up‐regulation of IGF2BP3 decreased the percentage of 5637 and J82 apoptotic cells. E, Western blotting analysis of IGF2BP3, Cyclin D1, Bax and Bcl2 expression after IGF2BP3 silenced. F, Western blotting analysis of IGF2BP3, Cyclin D1, Bax and Bcl2 expression after IGF2BP3 overexpressed. ****P < .0001

The role of IGF2BP3 in the cell cycle of bladder cancer cells was explored using flow cytometry assay. The flow cytometry assay showed a significant increase in the percentage of cells in the G0/G1 phase and a significant decreased that in the S phase after silencing of IGF2BP3. The converse was true after overexpression of IGF2BP3 in the cell lines. As shown in Figure 3A, cells in the G0/G1 phase were increased in IGF2BP3 knockdown UMUC3 and T24 cells compared to the control groups. IGF2BP3 overexpression promoted the cell cycle progression by raising the proportion of cells in the S phase in 5637 and J82 cells, compared to the vector control cells (Figure 3B). Moreover, Western blotting analysis revealed that cell cycle promotor cyclin D1 was down‐regulated after IGF2BP3 was silenced (Figure 3E). Meanwhile IGF2BP3 overexpressed cells showed the opposite trend (Figure 3F).

### 3.5 IGF2BP3 inhibits apoptotic activity in bladder cancer cells

As decreasing the IGF2BP3 expression greatly inhibited cell viability, it was hypothesized that IGF2BP3 may affect cell apoptosis in bladder cancer cells. To further investigate the mechanisms underlying the regulatory function of IGF2BP3 in the proliferation of bladder cancer cells, we performed flow cytometry assays with Annexin V and PI double staining in UMUC3, T24, 5637 and J82 cell lines. Flow cytometry disclosed that IGF2BP3 silencing significantly increased the percentage of apoptotic cells compared with the control group in T24 and UMUC3 cells, while the overexpression of IGF2BP3 yielded inverse results in 5637 and J82 cells. (Figure 3C and D). Furthermore, the expression level of cell apoptosis related biomarkers in the aforementioned cell lines was also examined. We found the Bcl2, an anti‐apoptotic factor, were significantly decreased whereas Bax, a pro‐apoptotic factor, was significantly increased after IGF2BP3 was silenced (Figure 3E). Meanwhile IGF2BP3 overexpressed cells showed the opposite trend (Figure 3F).

IGF2BP3 Promotes the Migration and Invasion in Bladder Cancer Cells.

Next, a transwell assay was performed to determine whether IGF2BP3 affected the migratory and invasive potential of bladder cancer cells. Firstly, the number of bladder cancer cells passed through the transwell membrane was observed to be reduced by siRNA‐mediated knockdown of IGF2BP3 in T24, suggesting that silencing IGF2BP3 could inhibit cell migration in bladder cancer (Figure S1A). Moreover, the transwell invasion assay also showed that silencing IGF2BP3 could significantly restrain the invasive ability of T24 cells (Figure S1A). At the same time, we found that overexpression IGF2BP3 significantly increased cell migration and invasion of 5637 cells (Figure S1B). These data showed that IGF2BP3 could promote the migration and invasion of bladder cancer cells.

### 3.6 IGF2BP3 regulates the tumorigenesis in vivo

**FIGURE 4:**
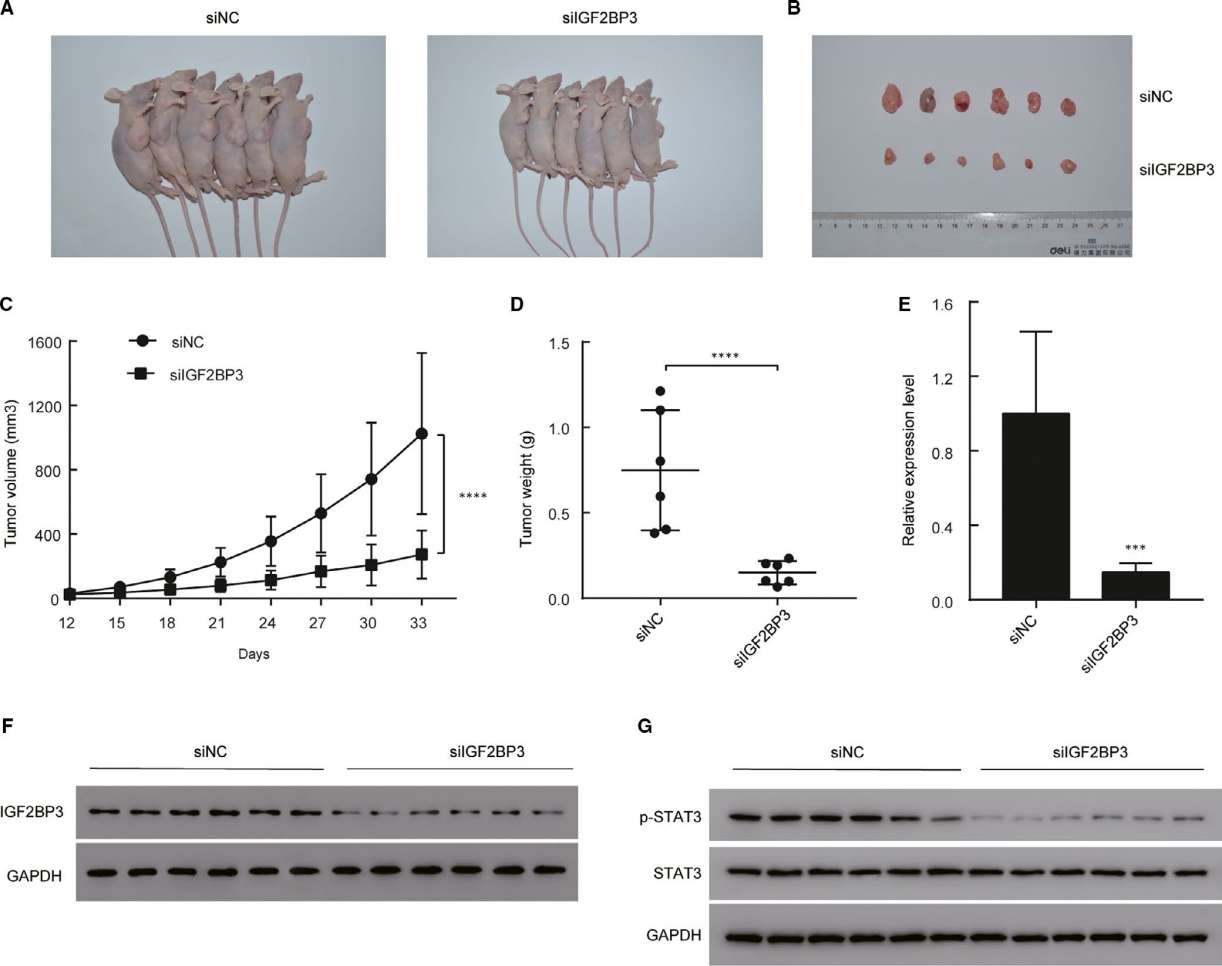
Overexpression of IGF2BP3 promoted the tumorigenesis of bladder cancer in vivo. A, Representative images of xenograft models. B, Xenograft tumours from respective groups were shown after injection with IGF2BP3 stable knockdown T24 cells and vector‐transfected T24 cells. C, Tumour growth curves were measured every three days. D, Average weight of excised tumours. E and F, RT‐qPCR and Western blotting analysis of IGF2BP3 expression in excised xenograft tumours. G, Western blotting analysis of p‐STAT3 and total STAT3 expression in excised xenograft tumours. ***P < .001, ****P < .0001

To verify the effects of IGF2BP3 on the tumorigenicity in vivo, xenograft models were established by injecting stable knockdown IGF2BP3 T24 cells and vector‐transfected T24 cells into subcutaneous tissues of nude mice. All nude mice developed xenogeneic tumours at the injection site (Figure 4A). Tumour growth of IGF2BP3 silenced cells was slower than that of the vector‐transfected cells (Figure 4B). As shown in Figure 4C and D, down‐regulation of IGF2BP3 significantly decreased the xenograft tumour volume and tumour weight compared to the control group. qRT‐PCR and Western blotting confirmed that IGF2BP3 expression levels were lower in the tumours injected with IGF2BP3 depleted cells than that in the vector‐transfected cells (Figure 4E and F). As shown in Figure 4G, Western blotting also showed that p‐STAT3 expression levels were lower in the tumours injected with IGF2BP3 depleted cells than that in the vector‐transfected cells. Thus, JAK/STAT3 pathway was activated in control group xenograft tumours but attenuated in siIGF2BP3 xenografts. Taken together, these results indicate that IGF2BP3 plays a vital role in the tumorigenicity of bladder cancer in vivo.

### 3.7 IGF2BP3 promoted the proliferation by regulating the JAK/STAT signalling pathway

**FIGURE 5:**
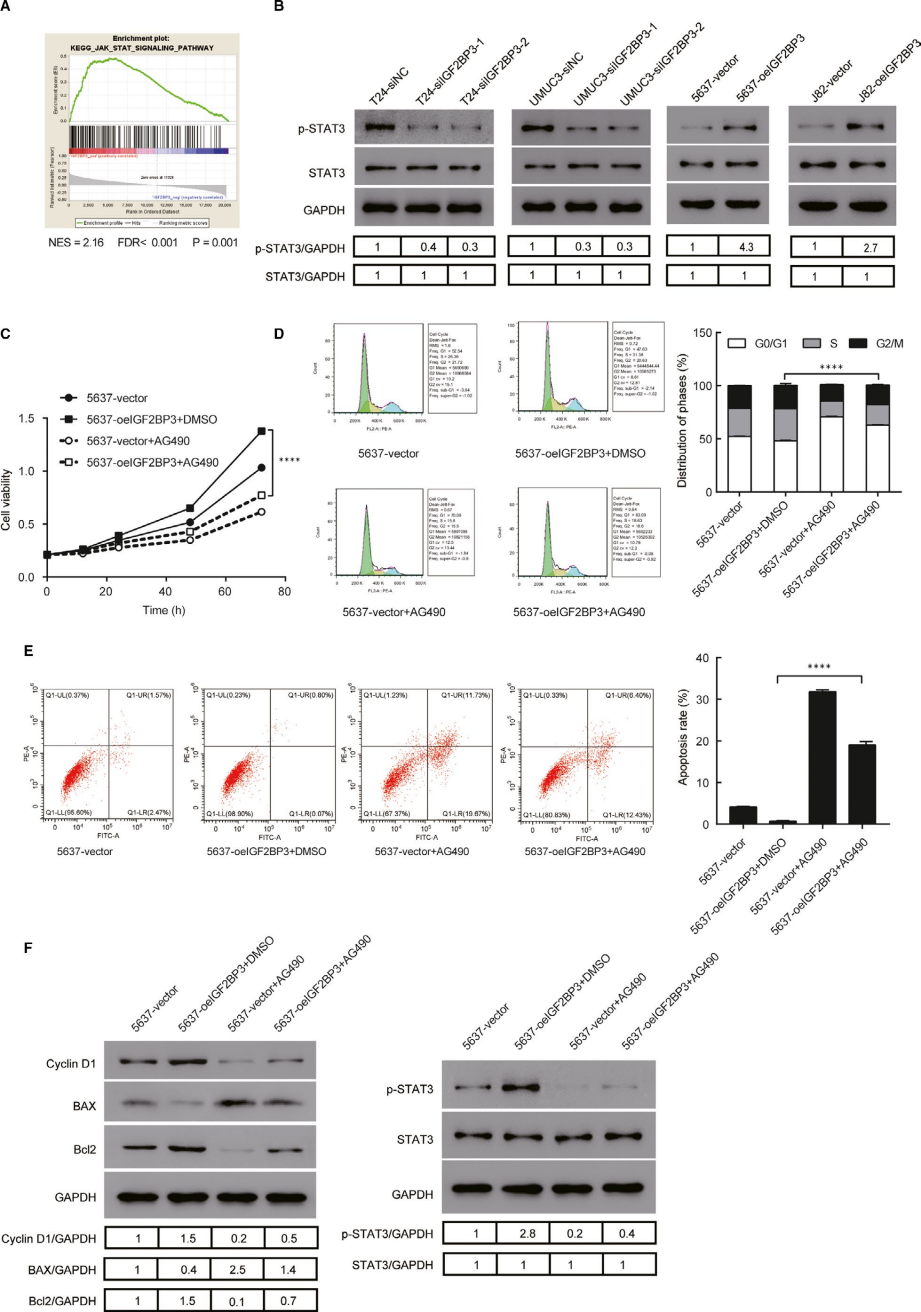
Regulation of JAK/STAT signalling by IGF2BP3. A, GSEA plot showing IGF2BP3 expression was positively associated with JAK/STAT pathway. B, Expression of JAK/STAT pathway key genes were detected by Western blotting. C, CCK‐8 assay revealed the role of JAK/STAT in the proliferation of IGF2BP3‐transfected cells. D, Flow cytometry assay revealed the role of JAK/STAT in the G1/S phase transition of IGF2BP3‐transfected cells. E, Flow cytometry assays with Annexin V and PI revealed the role of JAK/STAT in the apoptosis of IGF2BP3‐transfected cells. F, Western blotting revealed the role of JAK/STAT in the downstream cell cycle and cell apoptosis associated genes of IGF2BP3‐transfected cells. ****P < .0001

By performing GSEA analysis in the TCGA database, we found that IGF2BP3 expression was strongly associated with the JAK/STAT signalling pathway, which plays an essential role in cell proliferation.^16^ The results suggested that the JAK/STAT signalling pathway may be involved in the function of IGF2BP3 (Figure 5A). As shown in Figure 5B, phosphorylation of STAT3 was increased in IGF2BP3 overexpressed J82 and 5637 cells compared with the vector group but decreased in IGF2BP3 silenced T24 and UMUC3 cells. To confirm the role of the JAK/STAT pathway in the process of IGF2BP3 mediated cell proliferation, IGF2BP3 overexpressed 5637 cells were treated with 10umol/L AG490, a JAK/STAT pathway inhibitor, or vehicle (DMSO), for 24 hours. As shown in Figure 5C‐E, AG490 could inhibit the effect of IGF2BP3 on cell proliferation, cell cycle and cell apoptosis. Besides, the effect of IGF2BP3 on cell migration and invasion was reversed by AG490 (Figure S1C). Moreover, the inhibition of JAK/STAT rescued the effects of IGF2BP3 on cyclin D1, Bax, Bcl2 and phosphorylation of STAT3 (Figure 5F). Furthermore, through immunohistochemical studies of tissue microarrays, we assessed the expression of IGF2BP3 and phosphorylation of STAT3 in bladder cancer tissues (Figure S2A). As the Figure S2B shown, the expression of IGF2BP3 and phosphorylation of STAT3 showed significant positive correlation in bladder cancer tissues (*r* = 0.35, *P* = 0.029). These results suggest that IGF2BP3 enhances the cell proliferation via activation of the JAK/STAT pathway in bladder cancer cells.

## 4 DISCUSSION

Previous studies indicated that IGF2BP3 involved in the growth, chemo‐resistance and progression in many cancers.^17^, ^18^, ^19^, ^20^ IGF2BP3 has been identified as a positive regulator of cell proliferation and metastasis. A study by Xu et al revealed that IGF2BP3 was up‐regulated in colorectal cancer and associated with worse clinical outcome, which implies that IGF2BP3 harbours prognostic significance (14). IGF2BP3 overexpression has been linked to advanced disease stage and adverse clinical outcome in several cancers.^21^, ^22^, ^23^ Furthermore, IGF2BP3 was found to act as an oncogenic factor promoting proliferation and invasion of glioblastoma via activating PI3K/MAPK pathway.^24^ However, the role of IGF2BP3 in driving proliferation of bladder cancer has yet be elucidated.

In our study, we found IGF2BP3 could promote tumorigenesis of bladder cancer by JAK/STAT signalling. Thus, we have identified IGF2BP3 as a bladder‐specific pro‐proliferation marker that activates JAK/STAT pathway. Through an analysis of the TCGA database, we showed IGF2BP3 was up‐regulated in bladder cancer tissues, which was confirmed in experiments conducted with 5 pairs of bladder cancer tissues and bladder cancer cell lines. In the present study, results of clinically relevant study suggested that bladder cancer patients with higher IGF2BP3 levels were more prone to poor overall survival, and higher IGF2BP3 expression was correlated with a significantly increased risk of death in bladder cancer patients.

In order to confirm the role of IGF2BP3 in modulating tumour cell fate, IGF2BP3 was overexpressed or knocked down in bladder cancer cell lines. The results showed IGF2BP3 significantly inhibited cell apoptosis and promoted cell growth and cell cycle progression in vitro. The effects of IGF2BP3 on the tumorigenicity of bladder cancer cells were also confirmed by in vivo assays. IGF2BP3 silenced cells had a significantly decreased ability to form tumours in nude mice compared with vector‐transfected cells. Consistent with previous studies, our study demonstrated that IGF2BP3 could be considered to play an oncogenic role in the progression of bladder cancer by promoting cell growth.

Additionally, we further discovered IGF2BP3 promote cell growth of bladder cancer cells via JAK/STAT signalling. JAK/STAT signalling plays a crucial role in regulating cell growth, apoptosis and differentiation and is activated in many tumours.^25^, ^26^ The continuous activation of JAK/STAT could promote tumorigenesis.^27^ A previous study reported that lncRNA PART1 knocking down could inhibit proliferation, migration, and invasion via inactivating JAK/STAT signalling in non‐small cell lung cancer.^28^ Inhibition of JAK/STAT signalling suppresses cell growth and induces apoptosis, cell cycle arrest and inhibits cell invasion in colorectal cancer.^29^ Moreover, aberrant activated STAT3 was found in prostate cancer tissues but not in the normal tissues.^30^ Interleukin‐6 induces cell growth of prostate cancer by activating STAT3 signalling pathway.^31^ Our results showed that up‐regulation of IGF2BP3 could increase the expression of phosphorylation of STAT3 and IGF2BP3 knockdown significantly reduced the expression of phosphorylation of STAT3. According to the in vitro assays, we concluded that STAT3 affects phenotypes by regulating the cyclin D1, Bcl2 and Bax expression level. Then, we treated IGF2BP3 overexpressed cells with AG490, a JAK/STAT pathway inhibitor, or vehicle (DMSO) and found the inhibition of JAK/STAT rescued the effects of IGF2BP3 on phosphorylation of STAT3, cyclin D1, Bcl2 and Bax. Besides, the promoting effects of IGF2BP3 on cell proliferation were impaired by AG490, and the cell apoptosis rate of IGF2BP3 overexpressed cells cultured in AG490 was at partially increased as compared with IGF2BP3 overexpressed cells cultured in normal media. Moreover, percentage of cells in the S phase was significantly reduced in IGF2BP3 overexpressed cells with AG490 treatment compared with DMSO treatment. Therefore, we conclude that IGF2BP3 functions as a tumour promotor via JAK/STAT signalling pathway in bladder cancer development. In fact, it's normal for molecules to act through multiple pathways. It has been reported that overexpression of IGF2BP3 increased phosphorylation of IκBα and knockdown of IGF2BP3 reduced activation of IκBα in renal cell carcinoma (RCC) cells, indicating that IGF2BP3 activates the NF‐κB signalling pathway and promotes RCC cell migration and invasion.^32^ Ramaswamy Suvasini et al reported that IGF2BP3 as a glioblastoma‐specific pro‐proliferative and pro‐invasive marker acting through IGF‐2 resulting in the activation of oncogenic PI3K and MAPK pathways.^24^ The PI3K and the MAPK pathways, the downstream effectors of IGF‐2, are activated by IGF2BP3 and are found to be essential for IGF2BP3‐induced cell proliferation. It is also conceivable that IGF2BP3 has a multi‐pathway interaction function in regulating cell physiology and tumorigenesis in bladder cancer, which requires future investigations.

In summary, our study found IGF2BP3 up‐regulation exerted the positive biological role to promote the cell proliferation ability of bladder cancer cells in vitro and in vivo by modulating the JAK/STAT pathway. IGF2BP3 is a potential target for gene therapy of bladder cancer to make a better prognosis in the future.

## CONFLICT OF INTEREST

The authors confirm that there are no conflicts of interest.

## AUTHORS’ CONTRIBUTIONS

WH and GXW: conception and design of the research; YYL and CZ: acquisition of data; HHZ and JG: analysis and interpretation of data; XCZ and BF: statistical analysis; WH and YYL: drafting the manuscript; JG and GXW revision of manuscript for important intellectual content. All authors contributed to data analysis, drafting and revising the article, gave final approval of the version to be published and agree to be accountable for all aspects of the work. Wei Huang: Conceptualization (equal); Data curation (equal); Formal analysis (equal); Supervision (equal); Validation (equal); Writing‐original draft (equal); Writing‐review & editing (equal). Yuanyuan Li: Data curation (equal); Formal analysis (equal); Investigation (equal); Software (equal); Writing‐original draft (equal). Cheng Zhang: Data curation (equal); Investigation (equal); Methodology (equal). Huihai Zha: Investigation (equal); Methodology (equal); Software (equal). Xiaocheng Zhou: Formal analysis (equal); Validation (equal); Visualization (equal). Bin Fu: Project administration (equal); Visualization (equal). Ju Guo: Formal analysis (equal); Investigation (equal); Validation (equal); Writing‐review & editing (equal). Gongxian Wang: Conceptualization (equal); Project administration (equal); Resources (equal); Writing‐review & editing (equal).

## Supporting information

Fig S1

Fig S2

## Data Availability

The data used to support the findings of this study are available from the corresponding author upon request.
