# Supplementary figures and images for: IGF2BP3 facilitates cell proliferation and tumorigenesis via modulation of JAK/STAT signalling pathway in human bladder cancer
Source: J Cell Mol Med. 2020 Oct 22;24(23):13949–60. doi: 10.1111/jcmm.16003 (PMC7753985; doi:10.1111/jcmm.16003)

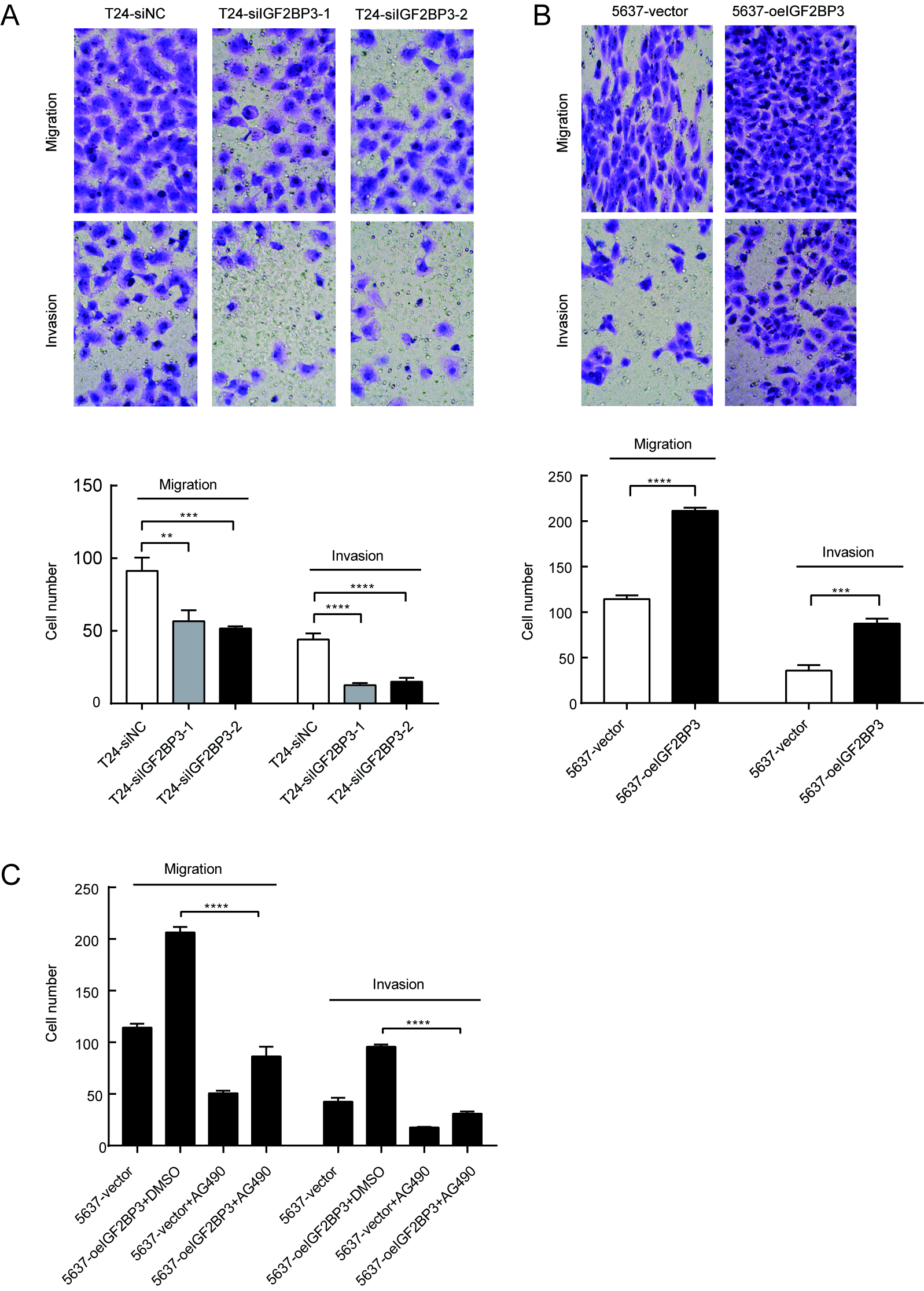

Supplement: Supplementary file 1 — Fig S1 [file JCMM-24-13949-s001.tif]

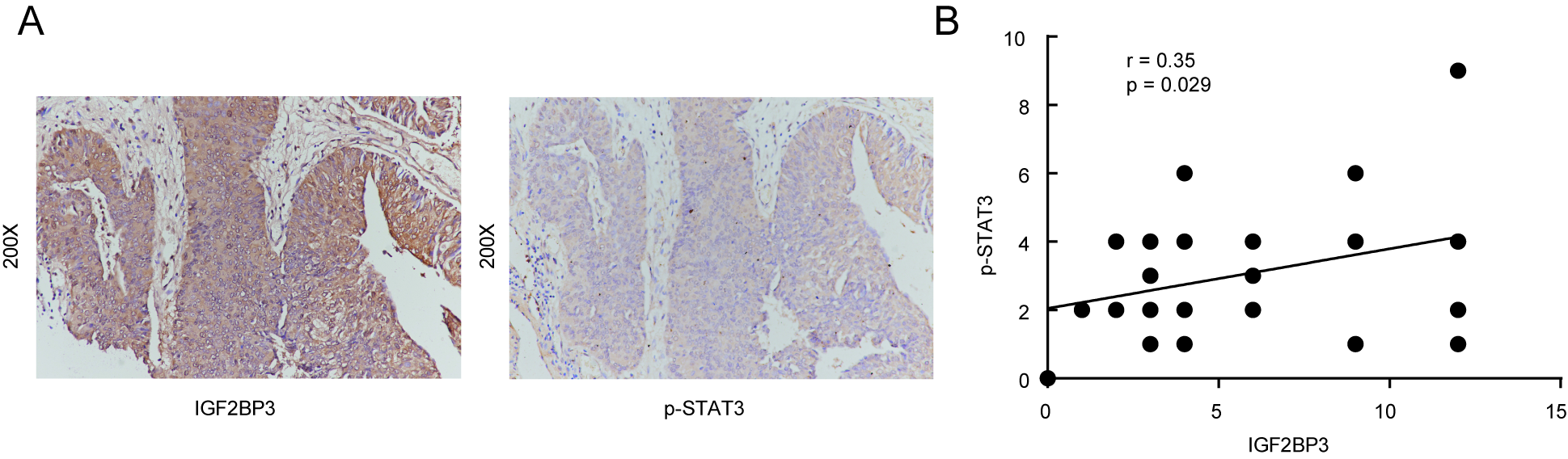

Supplement: Supplementary file 2 — Fig S2 [file JCMM-24-13949-s002.tif]
